# The serum uric acid to creatinine ratio as a diagnostic biomarker for normoalbuminuric diabetic kidney disease

**DOI:** 10.3389/fmed.2025.1584049

**Published:** 2025-05-14

**Authors:** Haoran Mu, Qilun Zhang, Wenyao Huang, Qiang Pan, Yan Zhang, Yanyan Lu, Zhangxiang Zhu, Xu Jiang, Guojuan Wang, Mao Zheng, Li Chen

**Affiliations:** ^1^Department of Endocrinology, The Third Affiliated Hospital of Anhui Medical University (The First People's Hospital of Hefei), Hefei, Anhui, China; ^2^Department of Central Laboratory, The Third People's Hospital of Bengbu Affiliated to Bengbu Medical University (Bengbu Central Hospital), Bengbu, Anhui, China; ^3^Department of Health Service Management, Anhui Medical University, Hefei, Anhui, China; ^4^Department of Laboratory, The Third Affiliated Hospital of Anhui Medical University (The First People’s Hospital of Hefei), Hefei, Anhui, China; ^5^Department of Endocrinology, The First Affiliated Hospital of USTC, Division of Life Sciences and Medicine, University of Science and Technology of China, Hefei, Anhui, China

**Keywords:** glomerular filtration rate, normoalbuminuric diabetic kidney disease, serum uric acid creatinine ratio, type 2 diabetes mellitus, urinary albumin creatinine ratio

## Abstract

**Background:**

To evaluate the potential of the serum uric acid to serum creatinine ratio (SUA/SCr) as a diagnostic biomarker for normoalbuminuric diabetic kidney disease (NADKD).

**Methods:**

We retrospectively analyzed demographic and biochemical data from 3,101 type 2 diabetes patients. Patients were stratified into non-diabetic kidney disease (non-DKD), albuminuric diabetic kidney disease (ADKD), and NADKD groups according to their estimated glomerular filtration rate (eGFR), urinary albumin creatinine ratio (UACR), and urinary albumin excretion rate (UAER). We employed multivariate logistic regression analyses using a stepwise forward-LR method to develop a nomogram. Both area under the curve (AUC) from receiver operating characteristic (ROC), and calibration curves were employed to assess the predictive accuracy of the nomogram. A decision curve analysis (DCA) was conducted to assess the clinical utility of the nomogram.

**Results:**

SUA/SCr, along with glycosylated hemoglobin A1c (HbA1C) and fasting plasma glucose (FPG), showed significant associations with NADKD, both pre- and post-propensity score matching (PSM). Seven variables were incorporated into the risk nomogram. The calibration plots indicated strong agreement between predicted and observed outcomes in both training and validation cohorts. The NADKD risk model demonstrated robust performance, as evidenced by the AUC from ROC analysis and DCA.

**Conclusion:**

SUA/SCr is a significant and independent predictor of NADKD risk. The developed nomograms offer valuable tools for clinical decision-making, potentially enhancing diagnostic accuracy for NADKD in type 2 diabetes patients.

## 1 Introduction

Diabetes represents a significant public health challenge worldwide. Among its complications, diabetic kidney disease (DKD) stands out as a predominant microvascular issue that can progress to end-stage renal disease (ESRD) (1–5). Approximately 20–40% of diabetes patients with concurrent DKD face heightened risks of cardiovascular events, including sudden cardiac death, myocardial infarction, and diabetic cardiomyopathy (6–11). Classical DKD is characterized by glomerular hyperfiltration, microalbuminuria, overt proteinuria, and a progressive decline in the estimated glomerular filtration rate (eGFR), ultimately leading to ESRD (12, 13). The severity of albuminuria correlates with increased risks of cardiovascular disease, chronic kidney disease progression, and mortality (10).

In recent years, epidemiological studies have reported a growing prevalence of normoalbuminuric diabetic kidney disease (NADKD) in patients with type 2 diabetes (5, 14–18). Patients with NADKD face a high risk of mortality and vascular events (16, 19–22), particularly those under 65 years of age (23). Moreover, NADKD serves as a robust predictor of all-cause mortality in type 2 diabetes patients (16), underscoring the importance of early diagnosis and intervention.

Serum uric acid to serum creatinine ratio (SUA/SCr) assesses serum uric acid (SUA) levels after standardizing the degree of renal function and reflects the net production of uric acid which is significantly correlated with the metabolic syndrome (24, 25) and is regarded as a novel risk factor for cardiovascular disease (26, 27). Recent studies have found SUA/SCr to be an independent risk factor for DKD (28), which was associated with a future decline in renal function in diabetes patients (25, 29). In our previous study, we observed that within the normal urinary albumin creatinine ratio (UACR) group of type 2 diabetes patients, SUA/SCr was lower in those with reduced eGFR compared to those with normal eGFR (28). This led us to hypothesize that SUA/SCr could potentially serve as a practical biomarker for identifying NADKD. However, due to limitations in sample size and other factors, the relationship between SUA/SCr and NADKD was not fully elucidated. To address this gap, we have expanded the sample size and collected comprehensive clinical data and biochemical parameters of diabetes patients treated at the Third Affiliated Hospital of Anhui Medical University during 2010–2023 in current study.

## 2 Materials and methods

### 2.1 Patients and procedures

The inclusion criteria consisted of patients diagnosed with type 2 diabetes in accordance with the diagnostic and classification criteria of the American Diabetes Association (ADA) (2024 edition) (30). Exclusion criteria encompassed type 1 diabetes, gestational diabetes, other specific types of diabetes, malignant tumors, liver disease, primary renal disease, acute and chronic infections, severe cardiac abnormalities, cases with incomplete data and those taking medications which affect uric acid and urinary protein levels including uric acid lowering agents, diuretics, sodium-glucose cotransporter 2 inhibitors and finelidone.

### 2.2 Data collection

We selected possible risk factors and indicators associated with NADKD from previous studies (14, 19, 28, 31–33). So, demographic data, including age, gender, height, weight, smoking history, family history of diabetes (with at least one first-degree relative affected), duration of diabetes, history of hypertension, and previous medication use, were collected from the electronic medical record system of the Third Affiliated Hospital of Anhui Medical University.

Laboratory data comprised measurements of blood urea nitrogen (BUN), serum uric acid (SUA), serum creatinine (SCr), serum cystatin-C (SCysc), glycosylated hemoglobin A1c (HbA1C), fasting plasma glucose (FPG), triglycerides (TG), total cholesterol (TCH), high-density lipoprotein (HDL), low-density lipoprotein (LDL), alanine transaminase (ALT), and aspartate transaminase (AST). Blood samples were obtained following an 8–10 h overnight fast for biochemical analysis. These parameters were measured using standard enzymatic methods with the Beckman Coulter Unicel DxC800 system (Anhui SenaiChi Hospital Management Co., Ltd.). HbA1C was assessed using high-performance liquid chromatography with the Arkray HA-1880 system (Anhui Guoke Kangyi Medical Technology Co., Ltd.). Morning spot urine samples were collected for the urinary albumin creatinine ratio (UACR) test, estimated via the dry immunomarker scattering quantitative method using the Abbott AFINION 2 system (Anhui Shunkang Medical Equipment Co., Ltd.).

### 2.3 Definition, groups, and assignment

DKD was defined as a UACR ≥30 mg/g and/or an eGFR <60 mL/(min 1.73 m^2^) persisting for over 3 months, absent other renal impairment causes in type 2 diabetes patients (3, 10). NADKD was defined based on previous study (5, 17, 34–36) as: (1) eGFR <60 mL/(min 1.73 m^2^); (2) at least 2 instances of urinary protein excretion <20 μg/min within 6 months, or a UAER <30 mg/24 h (under normal antihypertensive drug use), or UACR <30 mg/g; (3) exclusion of acute kidney injury, other causes of reduced eGFR, and secondary renal diseases. Albuminuric diabetic kidney disease (ADKD) was defined as DKD with UACR ≥30 mg/g sustained for over 3 months, irrespective of eGFR status.

### 2.4 eGFR collaboration formula

The eGFR was calculated using the standard Chronic Kidney Disease Epidemiology (CKD-EPI) Collaboration formula (37):


$$\text{eGFR}=a\times {\left(\frac{\mathrm{SCr}}{b}\right)}^{c}\times {\left(\frac{\text{SCysc}}{0.8}\right)}^{d}\times {\left(0.995\right)}^{\mathrm{age}}$$

Note:

*a* value: 130 for women, 135 for men.

*b* value: 0.7 for women, 0.9 for men.

*c* value: −0.248 for women with SCr ≤ 0.7 mg/dL and −0.601 for women with SCr > 0.7 mg/dL; −0.207 for men with SCr ≤ 0.9 mg/dL and −0.601 for men with SCr > 0.9 mg/dL.

*d* value: −0.375 for SCysc ≤ 0.8 mg/dL and −0.711 for SCysc > 0.8 mg/dL.

### 2.5 Statistical analysis

To mitigate the influence and imbalance of variables such as age, gender, duration of diabetes, body mass index (BMI), family history of diabetes, history of hypertension, and smoking history, propensity score matching (PSM) was employed. The NADKD group was matched with the ADKD group using a 1:2 nearest neighbor matching method without replacement, with a caliper value set at 0.02. Balance of control variables was assessed before and after matching.

Statistical analyses were conducted using R version: 3.5.1 and IBM SPSS 26.0. Data with normal distribution were expressed as mean ± standard deviation, while non-normally distributed data were presented as median and interquartile range (IQR, 25–75%). Categorical variables were expressed as percentages or ratios. Differences between groups were analyzed using chi-square tests or t-tests for normally distributed data, and Kruskal-Wallis or Mann–Whitney U tests for non-normally distributed data. Binary logistic regression analyses were performed to evaluate the correlation between SUA/SCr and NADKD, adjusting for other covariates. Multivariate logistic regression analyses were conducted using a stepwise regression method (forward-LR) to construct the nomogram. Nomogram were developed using the “rms” package based on independent predictors, with calibration plots assessing calibration ability. Receiver operating characteristic (ROC) curves were generated, and the area under the curve (AUC) was calculated to evaluate discrimination. Decision curve analysis (DCA) was also utilized to assess nomogram performance. A *p-value of* <0.05 was considered statistically significant.

## 3 Results

### 3.1 Clinical characteristics of type 2 diabetes patients grouped according to the SUA/SCr tertiles

**Figure 1:**
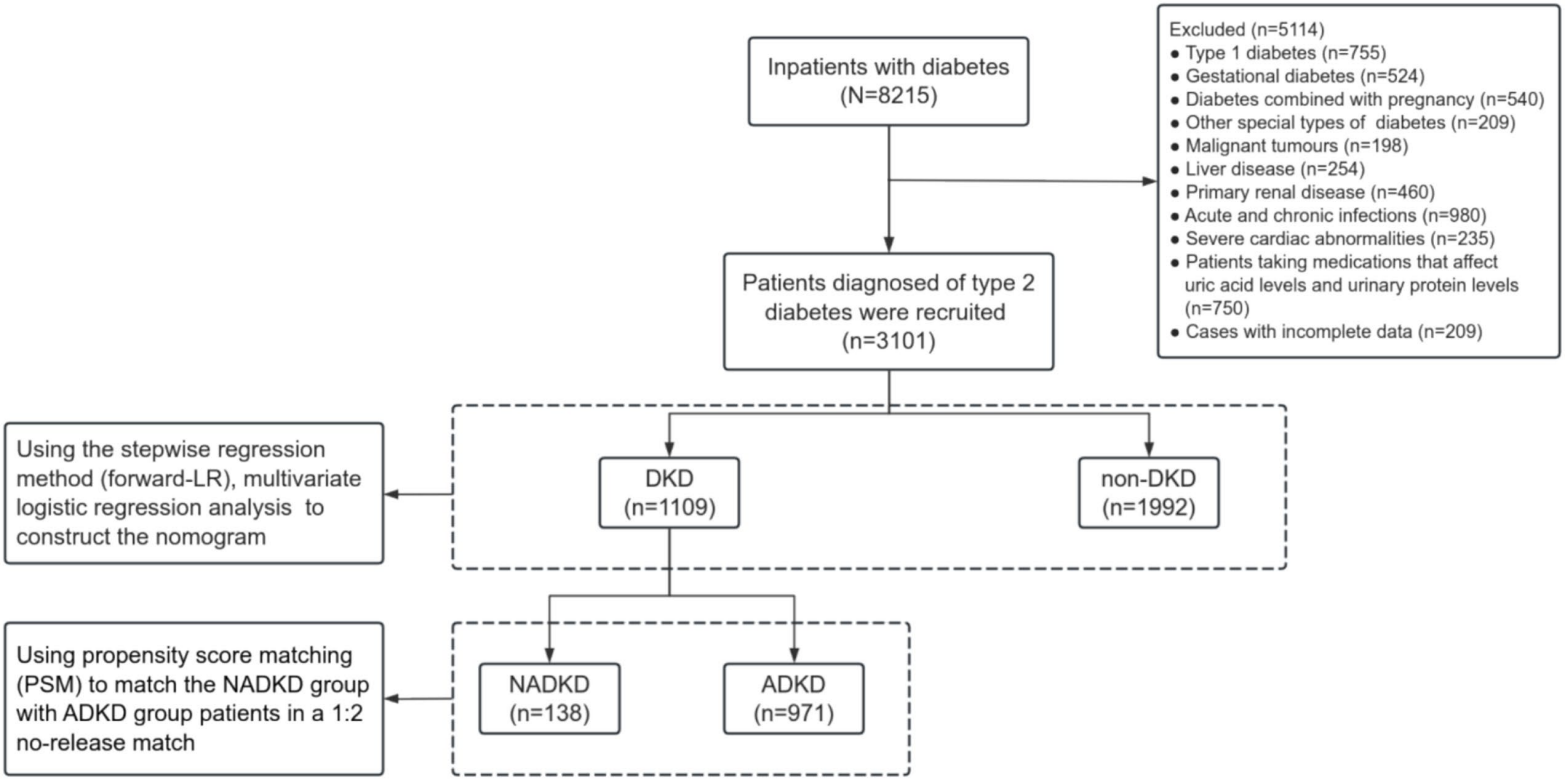
The workflow of this study.

We reviewed data from 3,101 patients with type 2 diabetes at the Third Affiliated Hospital of Anhui Medical University, spanning from 2010 to 2023 (Figure 1). Based on their tertiles of SUA/SCr, patients were divided into three groups of Tertile 1, Tertile 2, and Tertile 3 (Table 1). Many parameters, including age, gender, duration of diabetes, BMI, BUN, Cysc, HbA1c, and eGFR, as well as the proportions of HBP history and smoking history, showed significant differences between the three groups. The eGFR values were lowest in the Tertile 1 group and highest in the Tertile 3 group, accompanied by an SUA/SCr increase, and we also observed that the prevalence of NADKD were highest in the Tertile 1 group and lowest in the Tertile 3 group.

**Table 1:** Characteristics of T2DM patients according to the SUA/SCr tertiles.

Characteristics	Group			
	Tertile 1 (SUA/SCr ≤ 341.3)	Tertile 2 (341.3 < SUA/SCr ≤ 437.3)	Tertile 3 (SUA/SCr > 437.3)	*p*
Number, *n*	1.034	1.034	1.033	
Age, years	60(51.69)	57 (49.66)*	54 (44.63)*†	<0.001
Gender				<0.001
Male, *n*(%)	702 (67.9)	679 (65.7)	541 (52.4)*†	
Female, *n*(%)	332 (32.1)	355 (34.3)	492 (47.6)*†	
Duration of diabetes, months	84 (24.144)	60 (24.120)*	48 (12.120)*†	<0.001
BMI, kg/m^2^	23.8 (21.7, 25.7)	24.2 (22.1, 26.4)*	24.8 (22.8, 27.0)*†	<0.001
Diabetes family history, *n*(%)	363 (35.1)	376 (36.4)	361 (34.9)	0.762
History of hypertension, *n*(%)	503 (48.6)	460 (44.5)	432 (41.8)*	0.007
Smoking history, *n*(%)	370 (35.8)	366 (35.4)	307 (29.7)*†	0.005
BUN, mmol/L	5.5 (4.5, 6.8)	5.0 (4.1, 6.0)*	4.6 (3.9, 5.6)*†	<0.001
SCr (mg/dL)	0.9 (0.7, 1.1)	0.8 (0.6, 0.9)*	0.6 (0.5, 0.7)*†	<0.001
SUA (μmol/L)	255 (210, 312)	294 (251, 341)*	332 (283, 388)*†	<0.001
Cysc, mg/L	1.0 (0.8, 1.2)	0.9 (0.8, 1.0)*	0.8 (0.7, 1.0)*†	<0.001
HbA1c, %	9.0 (7.5, 10.2)	8.8 (7.2, 10.2)*	8.8 (7.1, 10.1)*	<0.001
FPG, mmol/L	8.4 (6.7, 10.9)	8.6 (6.8, 10.8)	8.5 (6.8, 10.7)	0.949
eGFR, mL/min/1.73 m^2^	83.7 (62.7, 102.2)	95.9 (79.8, 109.6)*	105.2 (90.7, 119.2)*†	<0.001
NADKD, *n*(%)	70 (6.8)	47 (4.5)*	21 (2.0)*†	<0.001

Data are expressed as median (IQR, 25 to 75%) or n (%), *compared to the Tertile 1, *P<* 0.05; †compared to the Tertile 2, *P <* 0.05. BMI, body mass index; BUN, blood urea nitrogen; SCysc, serum cystatin-C; HbA1C, glycosylated hemoglobin A1c; FPG, fasting plasma glucose; NADKD, normoalbuminuric diabetic kidney disease.

### 3.2 The correlation between SUA/SCr and eGFR

**Figure 2:**
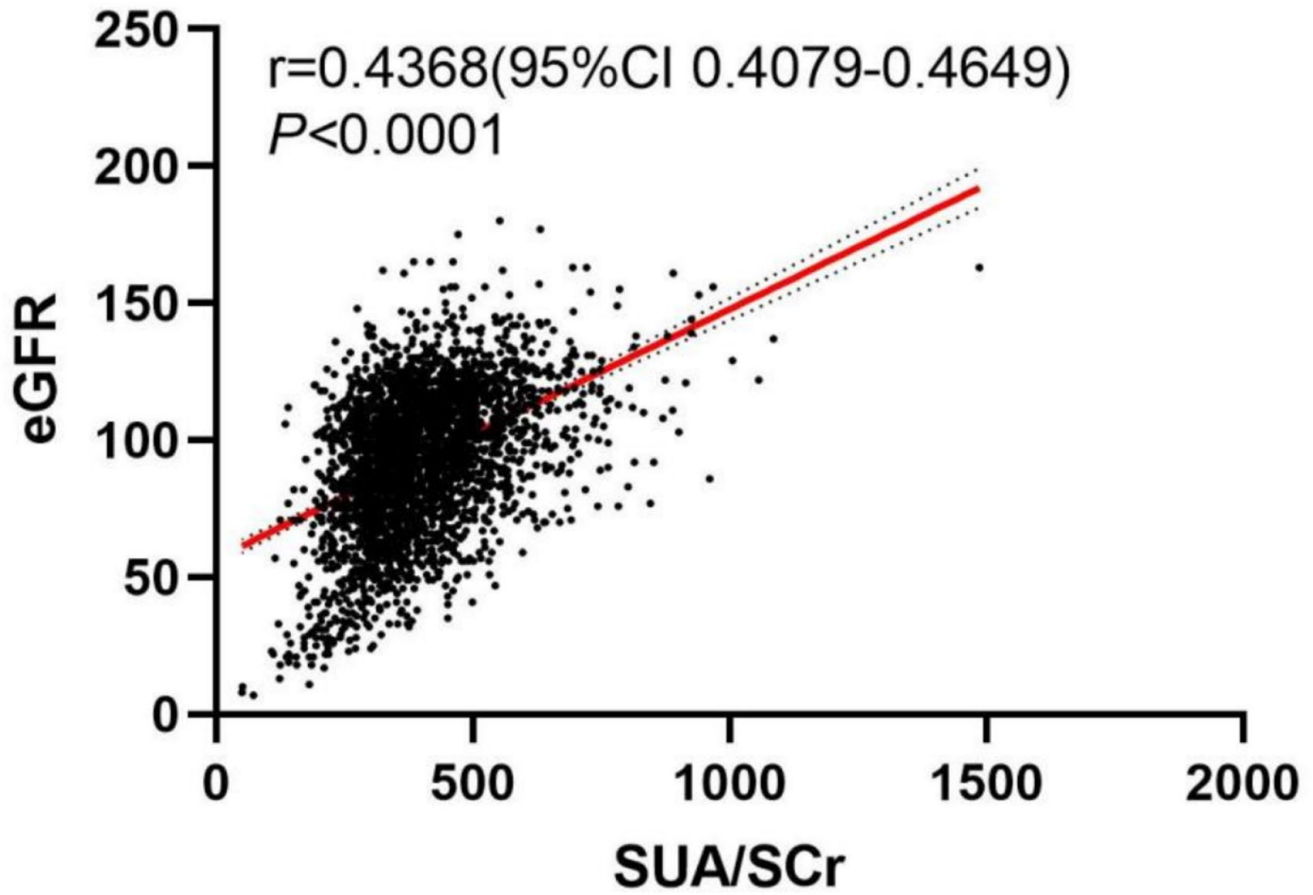
The scatter plot between SUA/SCr and eGFR.

To explore the relationship between SUA/SCr and eGFR, we conducted a visual analysis using a scatter plot. Figure 2 displays the scatter plot of SUA/SCr versus eGFR. From the plot, it is evident that the data points exhibit a clear upward trend (*p* < 0.0001, *r* = 0.4368).

### 3.3 Clinical characteristics of NADKD and ADKD

**Figure 3:**
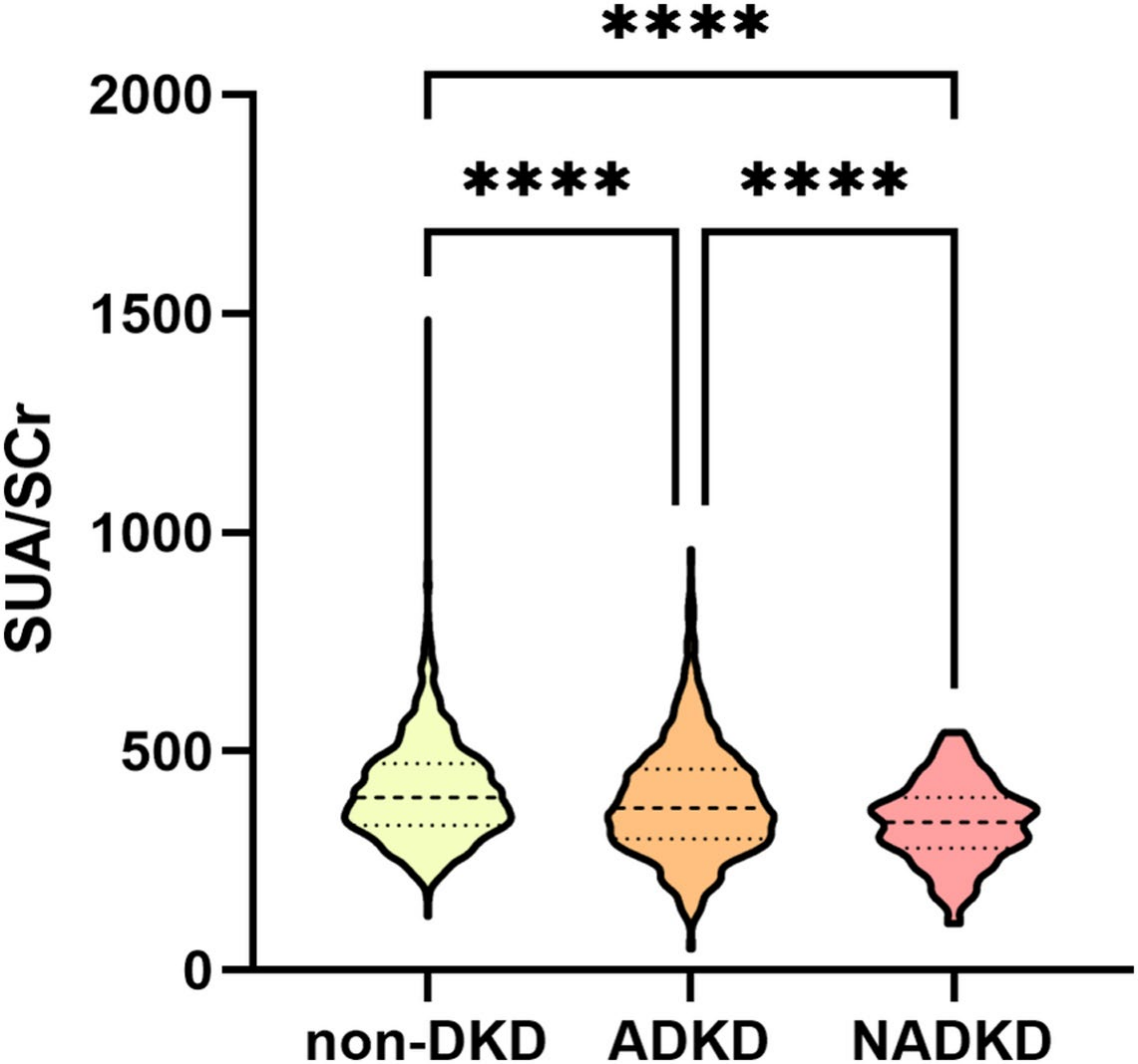
Violin plot of SUA/SCr between non-DKD, ADKD and NADKD groups.

Based on eGFR, UACR, or UAER, patients were classified into NADKD (*n* = 138), ADKD, (*n* = 971), and non-DKD (*n* = 1992) groups (Supplementary Table S1). NADKD accounted for 12.4% of all DKD cases (138/1109). As shown in Figure 3, SUA/SCr exhibits significant differences among three groups.

Post-matching analysis revealed that, compared to the ADKD group, the NADKD group had significantly lower levels of HbA1c, FPG, SUA/SCr, and eGFR (*p* < 0.05), while age, BUN, SUA, SCr, and Cysc were significantly higher (*p* < 0.05) (Table 2).

**Table 2:** Baseline clinical characteristics before and after propensity score matching between the NADKD and ADKD groups.

Characteristics	Entire cohort					PSM cohort			
	Non-DKD *n* = 1992	NADKD *n* = 138	ADKD *n* = 971	SMD	*P*-value	NADKD *n* = 117	ADKD *n* = 211	SMD	*P*-value
Age (years)	55 (47.64)	71 (64.77)	60 (50.69)	0.970	<0.001	70 (61.75)	68 (62.74)	0.141	0.222
Female, *n*(%)	738 (37.0)	63 (45.7)	378 (38.9)	0.136	0.156	54 (46.2)	97 (46.0)	0.004	1.000
Duration of diabetes (months)	60 (12.120)	90 (24.120)	96 (36.156)	0.092	0.302	96 (24.144)	96 (36.144)	0.022	0.842
BMI (kg/m^2^)	24.0 (22.1, 26.1)	25.0 (22.8, 27.1)	24.5 (22.3, 27.0)	0.111	0.223	24.8 (22.5, 27.2)	24.4 (22.5, 26.6)	0.044	0.706
Diabetes family history, *n*(%)	711 (35.7)	41 (29.7)	348 (35.8)	0.131	0.188	38 (32.5)	70 (33.2)	0.015	0.995
History of hypertension, *n*(%)	717 (36.0)	99 (71.7)	579 (59.6)	0.257	0.008	83 (70.9)	142 (67.3)	0.079	0.578
Smoking history, *n*(%)	676 (33.9)	26 (18.8)	341 (35.1)	0.373	<0.001	25 (21.4)	38 (18.0)	0.085	0.553
ALT (mmol/L)	21 (16.31)	18 (14.30)	20 (15.30)		0.083	18 (13.28)	20 (15.27)		0.171
AST (mmol/L)	19 (16.24)	20 (16.26)	19 (16.24)		0.201	19 (16.25)	19 (16.24)		0.522
BUN (mmol/L)	4.9 (4.0.5.8)	6.1 (4.8.8.0)	5.3 (4.2.6.7)		<0.001	6.3 (4.9.8.1)	5.6 (4.5.7.0)		0.004
SCr (mg/dL)	0.7 (0.6.0.8)	1.0 (0.9.1.3)	0.8 (0.6.1.0)		<0.001	1.0 (0.9.1.3)	0.8 (0.6.1.1)		<0.001
SUA (μmol/L)	285 (235.338)	345 (293.414)	309 (255.369)		<0.001	343 (282.413)	303 (251.353)		<0.001
Cysc (mg/L)	0.8 (0.7, 1.0)	1.5 (1.3, 1.7)	1.0 (0.8, 1.2)		<0.001	1.5 (1.3, 1.7)	1.0 (0.9, 1.3)		<0.001
TG (mmol/L)	1.5 (1.0, 2.3)	1.6 (1.0, 2.2)	1.8 (1.1, 2.7)		0.045	1.6 (1.0, 2.2)	1.6 (1.1, 2.4)		0.513
HbA1c (%)	8.7 (7.1, 10.1)	7.7 (6.8, 9.3)	9.1 (7.7, 10.7)		<0.001	7.8 (6.9, 9.3)	9.0 (7.5, 10.6)		<0.001
FPG (mmol/L)	8.3 (6.7, 10.5)	7.4 (6.1, 8.6)	9.1 (7.1, 11.4)		<0.001	7.3 (6.0, 8.7)	8.7 (6.7, 11.3)		<0.001
eGFR (mL/min/1.73 m^2^)	100.9 (86.3, 114.0)	53.7 (47.3, 57.1)	87.0 (64.6, 105.9)		<0.001	53.2 (47.1, 57.0)	78.3 (58.2, 96.5)		<0.001
SUA/SCr	410 ± 118	338 ± 92	387 ± 130		<0.001	332 ± 93	369 ± 130		0.031

Data are expressed as mean ± SD, median (IQR, 25–75%) or n (%). NADKD, normoalbuminuric diabetic kidney disease; ADKD, albuminuric diabetic kidney disease; BMI, body mass index; ALT, alanine transaminase; AST, aspartate transaminase; BUN, blood urea nitrogen; SCr, serum creatinine; SUA, serum uric acid; SCysc, serum cystatin-C; TG, triglyceride; HbA1C, glycosylated hemoglobin A1c; FPG, fasting plasma glucose; SUA/SCr, serum uric acid to serum creatinine ratio.

### 3.4 SUA/SCr as an independent risk predictor for NADKD

In our logistic regression analysis, we identified SUA/SCr as significantly associated with NADKD, even after adjusting for potential confounders (Table 3). Through stepwise regression, we further identified seven independent risk predictors for NADKD: age, HbA1c, BUN, SUA/SCr, BMI, duration of diabetes, and smoking history (Table 4). According to the results in the Supplementary Table S2, the P-value for SUA/SCr in the multivariable logistic regression is greater than 0.05, so SUA/SCr cannot be used as a diagnostic factor for ADKD (Supplementary Table S2).

**Table 3:** Correlation between SUA/SCr and NADKD in type 2 diabetes patients.

	Regression coefficient	SE	Wald-value	*P*-value	OR(95%CI)
Model 1	−0.005	0.001	22.353	<0.001	0.995 (0.993–0.997)
Model 2	−0.005	0.001	22.034	<0.001	0.995 (0.993–0.997)
Model 3	−0.006	0.001	26.798	<0.001	0.994 (0.991–0.996)
Model 4	−0.007	0.001	28.671	<0.001	0.993(0.991–0.996)

Model 1: no parameter was adjusted; Model 2: adjusted for age, gender; Model 3: Model 2 plus duration of diabetes, BMI, diabetes family history, history of hypertension, smoking history, FPG, HbA1C; Model 4: Model 3 plus hepatic function and serum lipids. SUA/SCr, serum uric acid to serum creatinine ratio; NADKD, normoalbuminuric diabetic kidney disease; BMI, body mass index; FPG, fasting plasma glucose; HbA1C, glycosylated hemoglobin A1c.

**Table 4:** Univariate and multivariate logistic analyses associated with NADKD.

Variable		Univariate analysis			Multivariate analysis	
	OR	95%CI	*P*-value	OR	95%CI	*P*-value
Age	1.114	1.094–1.134	<0.001	1.117	1.095–1.140	<0.001
HbA1c	0.834	0.762–0.913	<0.001	0.829	0.751–0.909	<0.001
BUN	1.235	1.156–1.319	<0.001	1.126	1.050–1.208	<0.001
SUA/SCr	0.995	0.993–0.996	<0.001	0.996	0.994–0.998	<0.001
BMI	1.061	1.011–1.113	0.017	1.128	1.070–1.189	<0.001
Duration of diabetes	1.002	1.001–1.004	0.022	0.996	0.994–0.999	0.001
Diabetes family history						
Yes	Re			Re		
No	0.760	0.523–1.103	0.149	1.086	0.719–1.641	0.694
History of hypertension						
Yes	Re			Re		
No	1.081	0.730–1.610	0.705	1.292	0.851–1.962	0.230
Smoking history						
Yes	Re			Re		
No	2.251	1.460–3.472	<0.001	1.629	1.025–2.590	0.039
Gender						
Male	Re			Re		
Female	0.719	0.510–1.014	0.060	0.814	0.534–1.240	0.338

NADKD, normoalbuminuric diabetic kidney disease; HbA1C, glycosylated hemoglobin A1c; BUN, blood urea nitrogen; SUA/SCr, serum uric acid to serum creatinine ratio; BMI, body mass index.

### 3.5 Diagnostic nomogram model establishment and validation

**Figure 4:**
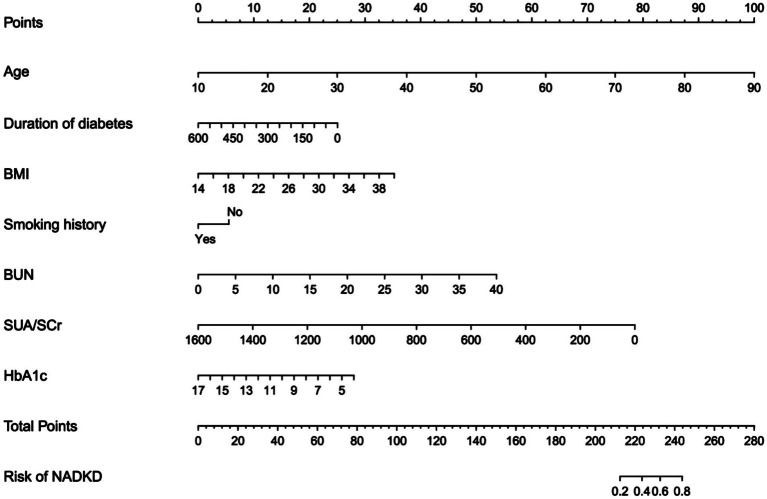
Diagnostic nomogram for the risk of NADKD. Nomogram including age, duration of diabetes (months), BMI, smoking history, BUN, SUA/SCr, HbA1c, and the risk of NADKD in type 2 diabetes patients. The nomogram allows the user to obtain the probability of the risk of NADKD corresponding to a patient’s combination of covariates.

**Figure 5:**
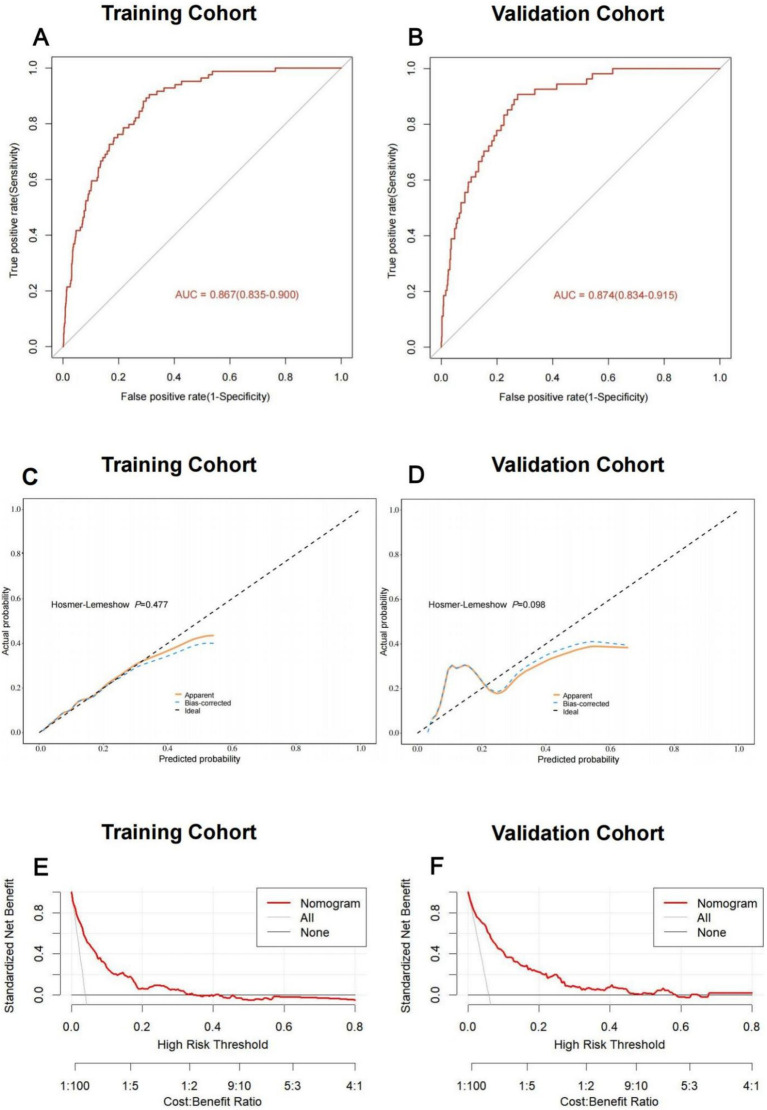
Discriminative ability and clinical usefulness of the predict model for the risk of NADKD. The ROC curves of nomogram in the training cohort **(A)**, validation cohort **(B)**. The calibration curves of nomogram in the training cohort **(C)**, validation cohort **(D)**. Actual the risk of NADKD is plotted on the y-axis; nomogram predicted probability for the risk of NADKD is plotted on the x-axis. The DCA curves of nomogram in the training cohort **(E)**, validation cohort **(F)**.

Using these independent predictors, we developed a diagnostic nomogram to assess NADKD risk (Figure 4). We randomly divided the 3,101 type 2 diabetes patients into training (70%) and validation cohorts (30%). Our ROC curves demonstrated AUC values of 0.867 (95% CI: 0.835–0.900) for the training cohort and 0.874 (95% CI: 0.834–0.915) for the validation cohort. In the training cohort, we found a cutoff value of 0.028, with sensitivity and specificity of 0.905 and 0.690, respectively, resulting in a Youden index of 0.595. For the validation cohort, the cutoff was 0.046, with sensitivity and specificity of 0.907 and 0.726, resulting in a Youden index of 0.633. The results demonstrate a consistent level of accuracy (Figures 5A,B). The calibration curve showed that the bias-corrected lines for the training and validation cohorts were close to the ideal lines (Figures 5C,D). DCA depicted the clinical net benefit achievable across varying risk thresholds (Figures 5E,F). The threshold ranges for DCA were derived from the training and validation cohorts based on the sensitivity and specificity of the model. Interventions were undertaken on patients whose assessed risk fell within the defined threshold range. Compared to the alternative of either intervening or not intervening on all patients, this approach yielded a superior net benefit.

## 4 Discussion

DKD is a prevalent complication of diabetes, traditionally characterized by the onset of proteinuria preceding a decline in GFR. However, recent studies have identified a subset of patients with diabetes who experience a reduction in GFR without proteinuria, termed NADKD (5, 13–16, 21, 38). These patients are at an elevated risk for mortality and vascular events (16, 19–23). Current diagnostic approaches, reliant on GFR and UAER/UACR, are influenced by various factors and present accessibility challenges (5, 17). Gold-standard GFR measurement techniques, such as inulin clearance and renal Emission Computed Tomography, are not routinely available in clinical settings. Instead, eGFR is commonly used but is also influenced by many factors and is less accurate in the early stages of DKD (38, 39). Additionally, urinary albumin levels can be affected by conditions such as fever, infection, and hypertension (10, 40, 41). Currently, biomarkers such as neutrophil gelatinase-associated lipocalin (NGAL), retinol binding protein (RBP), monocyte chemoattractant protein-1 (MCP-1) and kidney injury molecule-1 (KIM-1) play a significant role in the early diagnosis and monitoring of kidney damage progression and hold diagnostic value for NADKD (42, 43). However, these biomarkers are more expensive, have complex detection methods, are not widely used in clinical practice, and have limited applicability. Therefore, there is a pressing need for simple and practical biomarkers for NADKD prediction and diagnosis, particularly in resource-limited areas.

In this study, we retrospectively analyzed data from 8,215 diabetes patients at our center from 2010 to 2023, ultimately including 3,101 patients after stringent screening. PSM analysis revealed a significant difference in SUA/SCr between NADKD and ADKD groups. Logistic regression analysis confirmed the significant association of SUA/SCr with NADKD after adjusting for confounders. Multivariate analysis identified age, smoking history, HbA1c, BUN, diabetes duration, BMI, and SUA/SCr as independent predictors of NADKD. We constructed and validated a nomogram based on these factors to predict NADKD risk in type 2 diabetes patients, demonstrating acceptable predictive performance through ROC and DCA.

SUA is the most abundant aqueous antioxidant in the human body. It has antioxidant properties, protects against DNA damage, participates in redox reactions, and scavenges oxygen free radicals, contributing to over 50% of the antioxidant capacity in human blood circulation. On the other hand, SUA may also induce pro-oxidant stress, primarily within cells, potentially involving free radicals, nitric oxide, and myeloperoxidase. However, SUA levels are closely related to renal excretion. The SUA/SCr ratio, as a novel biomarker of kidney function, can be used to evaluate the next generation of SUA (24, 25).

The prevalence of NADKD within DKD in type 2 diabetes patients has been reported to range from 7 to 50% (14, 16–18, 31, 32, 34, 44). Consistent with these findings, our study found that NADKD constituted 12.4% of DKD cases among type 2 diabetes inpatients. Remarkably, the prevalence of NADKD varied widely across different studies, which may be attributed to the difference of inclusion criteria, geography, diet, ethnicity, evaluation formula of eGFR and other factors.

The present study demonstrated significantly lower HbA1c levels in NADKD patients compared to the ADKD group, which is in accordance with previous studies (16–19, 31, 33). This may be attributed to deterioration in renal function, which increases the risk of anemia in patients (10). Previous research has indicated that anemic patients have lower HbA1c levels (45). Furthermore, as the severity of anemia increases, the reduction in HbA1c becomes more pronounced.

The etiology of NADKD remains uncertain, with a number of potential factors implicated in its pathogenesis including vascular factors, inflammatory response factors, drug-related factors, and so forth (43, 46, 47). Classical renal structural changes were seen less frequently in NADKD patients (48). Interstitial fibrosis exerts a more pronounced impact on the deterioration of renal function than glomerular injury and is not influenced by the quantity of albumin present in the urine (49). NADKD is distinguished by a reduction in renal function that is not accompanied by the presence of albuminuria. SUA/SCr reflects net uric acid production, we observed that the level of SUA did not show an entirely consistent manifestation with SCr. This phenomenon may be theoretically explained by impaired renal tubules. So, we postulate that interstitial injury is associated with NADKD. Furthermore, in instances of extensive tubulo-interstitial injury, the reabsorption of SUA is reduced, resulting in an elevation of SCr and a decline in the SUA/SCr ratio (50, 51). The above evidence may explain the use of SUA/SCr as a biomarker and diagnostic marker for NADKD.

This study has some limitations. Firstly, being a single-center retrospective study, it establishes associations rather than causality. Secondly, while PSM analysis minimized bias, prospective studies are needed further elucidate the SUA/SCr-NADKD relationship. Thirdly, Multicenter clinical validation is necessary to assess the external utility of our nomograms. Finally, the pathogenesis of NADKD remains to be elucidated, and the need for further animal and cellular studies is evident.

## 5 Conclusion

In summary, SUA/SCr has been identified as an independent risk factor and a diagnostic indicator for NADKD. The nomogram developed from SUA/SCr demonstrated strong predictive performance. These tools can aid clinicians in accurately identifying high-risk patients, allowing for tailored interventions that can improve patient outcomes.

## Data Availability

The raw data supporting the conclusions of this article will be made available by the authors, without undue reservation.
