# Supplementary material for: The serum uric acid to creatinine ratio as a diagnostic biomarker for normoalbuminuric diabetic kidney disease
Source: Front Med (Lausanne). 2025 May 14;12:1584049. doi: 10.3389/fmed.2025.1584049 (PMC12116525; doi:10.3389/fmed.2025.1584049)
Supplement: Supplementary file 1 [file Table_1.docx]

Supplementary Material

**Table S1** Basic patient characteristics and biochemical indicators

| **Characteristics** | **Group** | | |
| --- | --- | --- | --- |
|  | **non-DKD** | **ADKD** | **NADKD** |
| Number, *n* | 1992 | 971 | 138 |
| Age (years) | 55(47,64)*† | 60(50,69)* | 71(64,77) |
| Gender |  |  |  |
| Male, *n*(%) | 1254(63.0) | 593(61.1) | 75(54.3) |
| Female, *n*(%) | 738(37.0) | 378(38.9) | 63(45.7) |
| Duration of diabetes (months) | 60(12,120)*† | 96(36,156) | 90(24,120) |
| BMI (kg/m^2^) | 24.0(22.1,26.1)*† | 24.5(22.3,27.0) | 25.0(22.8,27.1) |
| Diabetes family history, *n*(%) | 711(35.7) | 348(35.8) | 41(29.7) |
| History of hypertension, *n*(%) | 717(36.0)*† | 579(59.6)* | 99(71.7) |
| Smoking history, *n*(%) | 676(33.9)* | 341(35.1)* | 26(18.8) |

Data are expressed as median (IQR, 25 to 75%) or n (%), *compared to the NADKD group, P＜0.05; †compared to the ADKD group, P＜0.05;

Abbreviations: non-DKD, non-diabetic kidney disease; ADKD, albuminuric diabetic kidney disease; NADKD, normoalbuminuric diabetic kidney disease; BMI, body mass index.

**Table S2** Univariate and multivariate logistic analyses associated with ADKD

| Variables | Univariate analysis | | | | |  | Multivariate analysis | | | | |
| --- | --- | --- | --- | --- | --- | --- | --- | --- | --- | --- | --- |
|  | β | S.E | Z | *P* | OR (95%CI) |  | β | S.E | Z | *P* | OR (95%CI) |
| Smoking history |  |  |  |  |  |  |  |  |  |  |  |
| YES |  |  |  |  | 1.00 (Reference) |  |  |  |  |  | 1.00 (Reference) |
| NO | 0.04 | 0.08 | 0.48 | 0.634 | 1.04 (0.89 ~ 1.21) |  | -0.09 | 0.09 | -1.06 | 0.291 | 0.91 (0.77 ~ 1.08) |
| Age | -0.04 | 0.00 | -11.73 | <.001 | 0.96 (0.96 ~ 0.97) |  | -0.03 | 0.00 | -8.06 | <.001 | 0.97 (0.96 ~ 0.98) |
| Duration of diabetes | -0.01 | 0.00 | -11.45 | <.001 | 0.99 (0.99 ~ 0.99) |  | -0.01 | 0.00 | -7.20 | <.001 | 0.99 (0.99 ~ 0.99) |
| BMI | -0.04 | 0.01 | -3.85 | <.001 | 0.96 (0.94 ~ 0.98) |  | -0.09 | 0.01 | -7.21 | <.001 | 0.91 (0.89 ~ 0.94) |
| BUN | -0.27 | 0.02 | -11.70 | <.001 | 0.76 (0.73 ~ 0.80) |  | -0.21 | 0.02 | -8.59 | <.001 | 0.81 (0.77 ~ 0.85) |
| HbA1c | -0.08 | 0.02 | -4.49 | <.001 | 0.92 (0.89 ~ 0.96) |  | -0.13 | 0.02 | -6.46 | <.001 | 0.88 (0.85 ~ 0.92) |
| SUA/SCr | 0.01 | 0.00 | 6.35 | <.001 | 1.01 (1.01 ~ 1.01) |  | 0.00 | 0.00 | 1.16 | 0.245 | 1.00 (1.00 ~ 1.00) |
| OR: Odds Ratio, CI: Confidence Interval | | | | | | | | | | | |

To further validate whether SUA/SCr can predict ADKD, we performed multivariate logistic regression. According to the results in the Table S2, the *P*-value for SUA/SCr in the multivariable logistic regression is greater than 0.05, so SUA/SCr cannot be used as a diagnostic factor for ADKD.
